# Why is multiple micronutrient powder ineffective at reducing anaemia among 12–24 month olds in Colombia? Evidence from a randomised controlled trial

**DOI:** 10.1016/j.ssmph.2016.02.004

**Published:** 2016-03-07

**Authors:** Alison Andrew, Orazio Attanasio, Emla Fitzsimons, Marta Rubio-Codina

**Affiliations:** a Institute for Fiscal Studies, 7 Ridgmount Street, London WC1E 7AE, UK; b University College London, London, UK; c UCL Institute of Education, London, UK; d Inter-American Development Bank, Washington DC, USA

**Keywords:** Anaemia, Iron-deficiency, Haemoglobin, Colombia, Micronutrients, Multiple micronutrient powder, Child, Nutrition

## Abstract

In Colombia’s bottom socio-economic strata, 46.6% of children under two are anaemic. A prevalence of above 20% falls within the WHO guidelines for daily supplementation with multiple micronutrient powder (MNP). To evaluate the effect of daily MNP supplementation on anaemia amongst Colombian children aged 12–24 months we ran a cluster RCT (*n*=1440). In previous work, we found the intervention had no impact on haemoglobin or anaemia in this population. In this current paper, we investigate this null result and find it cannot be explained by an underpowered study design, inaccurate measurements, low adoption of and compliance with the intervention, or crowding out through dietary substitution. We conclude that our intervention was ineffective at reducing rates of childhood anaemia because MNP itself was inefficacious in our population, rather than poor implementation of or adherence to the planned intervention. Further analysis of our data and secondary data suggests that the evolution with age of childhood anaemia in Colombia, and its causes, appear different from those in settings where MNP has been effective. Firstly, rates of anaemia peak at much earlier ages and then fall rapidly. Secondly, anaemia that remains after the first year of life is relatively, and increasingly as children get older, unrelated to iron deficiency. We suggest that factors during gestation, birth, breastfeeding and early weaning may be important in explaining very high rates of anaemia in early infancy. However, the adverse effects of these factors appear to be largely mitigated by the introduction of solid foods that often include meat. This renders population wide MNP supplementation, provided after a diet of solid foods has become established, an ineffective instrument with which to target Colombia’s childhood anaemia problem.

## Highlights

• In contrast to other studies, MNP did not increase haemoglobin in Colombian infants.
• We conclude that MNP was both ineffective and inefficacious in our population.
• This is likely due to two differences from populations where MNP has been successful.
• First, rates anaemia in Colombia peak at earlier ages (6–12 months) and fall quicker.
• Second, anaemia is less (and increasingly less with age) related to iron deficiency.

## Introduction

Multiple micronutrient powder (MNP) has been described as an effective and scalable way to combat childhood and infant anaemia. The WHO, for instance, recommends population wide daily MNP supplementation in settings where anaemia has a prevalence of 20% or greater amongst under-twos. Anaemia affects nearly half (46.6%) of under-twos in Colombia’s lowest socio-economic strata. This rises to nearly 70% of 6–12 month olds (own estimates using ENSIN 2010 data (Instituto Colombiano de Bienestar Familiar, 2015)). Colombia in general, and its youngest and most disadvantaged children in particular meet the WHO’s criteria for MNP adoption. In an earlier paper (Attanasio et al., 2014) we evaluated a daily MNP supplementation intervention targeted at children aged 12–24 months from the poorest households in Colombian small towns. We found no effect on childhood anaemia. In this paper, we investigate why MNP supplementation had no impact.

### Early childhood anaemia

Anaemia refers to a sub-optimally low concentration of haemoglobin (Hb) in the blood which reduces its capacity to transport oxygen. Very severe anaemia in young children probably increases mortality rates (Brabin, Premji, & Verhoeff, 2001) while less severe cases contribute to delays in children’s cognitive and motor development (Grantham-McGregor and Ani, 2001, Thompson et al., 2013).

Deficiencies of iron and other micronutrients, arising from inadequate intake or poor absorption, are the most prominent causes of anaemia in young children (Balarajan, Ramakrishnan, Ozaltin, Shankar, & Subramanian, 2011). Rapid growth at the start of life means infants’ requirements for micronutrients are high, especially relative to body size and energy intake, whilst their stores are low (World Health Organization, 2005). Coupled with often unvaried diets, this leaves many young children extremely vulnerable to nutritional anaemia. Iron deficiency is widely reported to account for approximately half the cases of childhood anaemia worldwide (DeMaeyer and Adiels-Tegman, 1985), although the generalisability of this result is not well established (Stoltzfus, 2001). Aside from micronutrient deficiencies, other significant causes of childhood anaemia are parasitic infections and malaria, although the latter is not relevant in our regions of Colombia.

### Multiple micronutrient powder

Multiple micronutrient powder (MNP) was designed and described as a cost-effective and scalable way to target nutritional anaemia (Zlotkin et al., 2005). Single dose sachets of powder, typically containing iron, zinc, vitamin A and other vitamins and minerals, are sprinkled over a child’s food immediately prior to consumption. Because MNP contains multiple micronutrients it can simultaneously address different nutritional causes of anaemia and could, therefore, be more effective than iron supplementation alone. It is argued to be superior to tablets or drops through overcoming problems of poor adherence (Zlotkin et al., 2005).

**Fig. 1:**
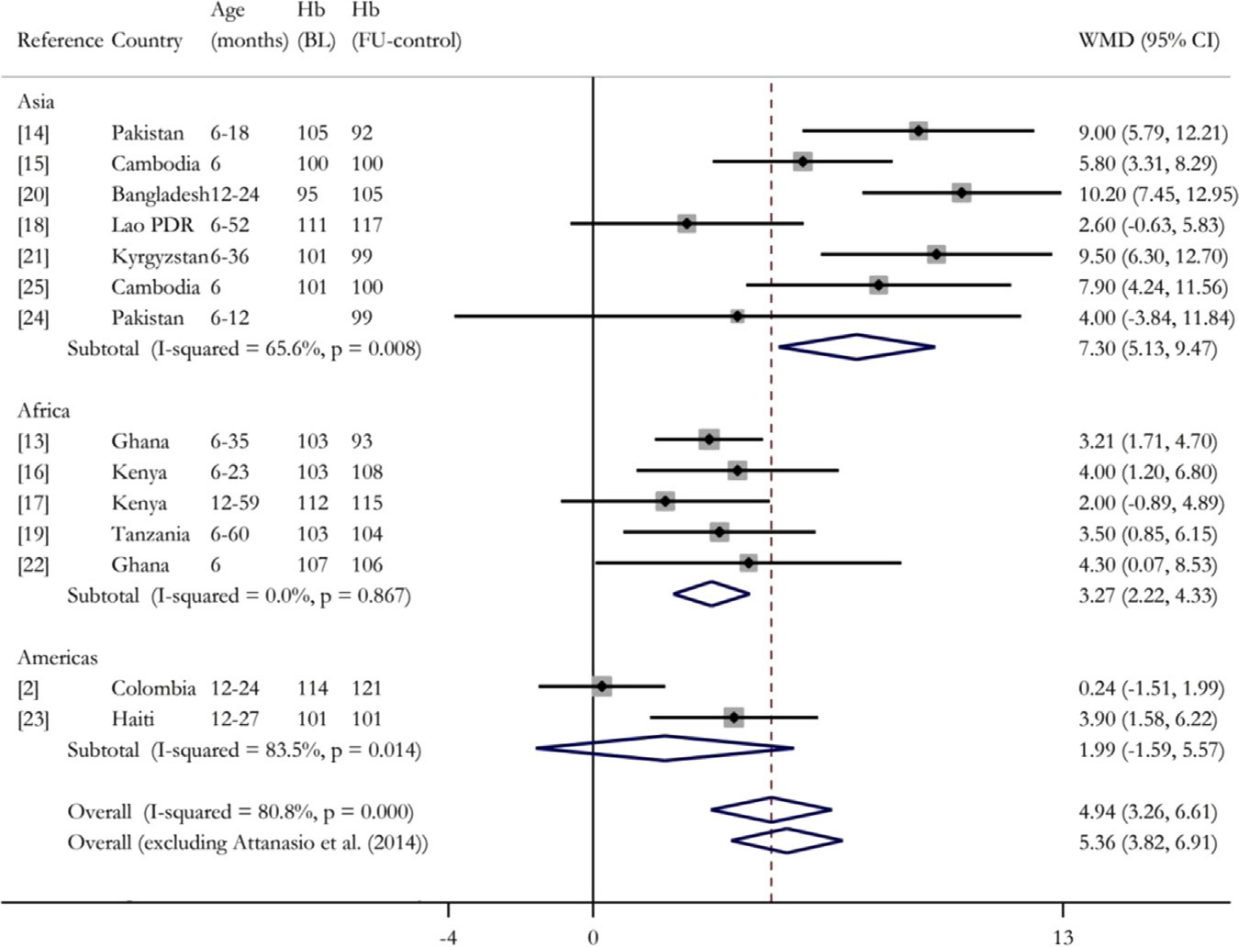
Meta analysis of the effect of MNP interventions on mean haemoglobin. Random effects meta-analysis performed using STATA’s metan command. Age(months)=age in months at the start of intervention. WMD=Weighted Mean Difference. Hb(BL)=baseline haemoglobin (g/L). Hb(FU-control)=haemoglobin (g/L) of the control group at follow-up. Bottom row excludes our study. Studies weighted using effective sample size accounting for design effects in cluster randomised controlled trials. For studies that do not report the intra-cluster correlation coefficient (ICC) we use 0.07 (mid point between the two studies that report an ICC: Attanasio et al. (2014) and Jack et al. (2012)) to calculate the effective sample size. Details of all studies provided in Table A1.

The previous empirical evidence on MNP is encouraging. On the basis of a Cochrane systematic review (De-Regil, Suchdev, Vist, Walleser, & Peña-Rosas, 2011), the WHO (World Health Organization, 2011a) recommended daily MNP supplementation for all young children in populations with a prevalence of childhood anaemia (in children under 2 years) greater than 20%. The forest plot in Fig. 1 updates the evidence from that systematic review. It includes all published randomised trials found through an extensive but non-systematic search of literature published up to and including November 2015, which compare MNP with iron to no intervention, or a placebo, or MNP without iron, in populations of children under two years at the start of the intervention (Attanasio et al., 2014, Zlotkin et al., 2013, Soofi et al., 2013, Jack et al., 2012, Suchdev et al., 2012, Macharia-Mutie et al., 2012, Kounnavong et al., 2011, Veenemans et al., 2011, Lemaire et al., 2011, Lundeen et al., 2010, Adu-Afarwuah et al., 2007, Menon et al., 2007, Sharieff et al., 2006, Giovannini et al., 2006). Table A1 summarises each study. In ten of the thirteen studies (excluding ours) the mean haemoglobin of the group receiving MNP, controlling for baseline haemoglobin where available, was significantly higher than that of the control group. Overall, a random effects meta-analysis estimates a Weighted Mean Difference (WMD) in haemoglobin concentration across these studies of 5.36 g/L (95% CI: 3.82–6.91 g/L).

Although the evidence for MNP is generally positive, the studies are drawn from diverse populations in terms of geography, age and health (Table A1). The precise interventions also differed. Thus, we would expect substantial heterogeneity in effects between studies. Indeed, the estimated I-squared of 79.7% (excluding our trial) suggests that almost four fifths of the total variation between studies is due to heterogeneity, rather than sampling variation. In the context of meta-analyses of health interventions, this is high (Higgins, Thompson, Deeks, & Altman, 2003).

In general, the studies in Asia had substantially greater impacts on haemoglobin than those in Africa, although even within geographic regions the degree of heterogeneity is large (Fig. 1). On average, studies with high levels of haemoglobin at baseline recorded lower effect sizes. However, substantial heterogeneity remains within subgroups of studies with similar mean baseline haemoglobin.

In the face of significant heterogeneity, estimated WMD from meta-analyses are not particularly informative. They are poor predictors of the effect in any one context and estimate a mean effect for a poorly defined group of populations (those where studies have been carried out and results published). Therefore, these estimates, alone, are ill-suited to create guidelines that will be applied to broad populations. In response to this problem, Stoltzfus (2014) argues for moving beyond a ‘black box’ approach to understanding nutrition interventions and using mechanistic evidence to understand generalisability of results to other settings. This is what we attempt in this paper.

### Hypotheses

In this paper, we take the null result of our Colombian MNP intervention and the heterogeneity in the estimated effects of MNP reported by other studies as a starting point. After examining the accuracy of our measurements and the power of the study design, we further investigate the null result by considering three sets of hypotheses. First, we assess the hypotheses that MNP was effective for two subpopulations of children for whom we might anticipate larger impacts – children with lower concentrations of haemoglobin at baseline and younger children. Second, we explore whether ineffectiveness of MNP was due to poor implementation or indirect behavioural effects (i.e. whether MNP may still have been efficacious despite being ineffective). In particular, we examine whether no effects of MNP were detected because of either (i) low adoption of or compliance with the intervention or (ii) because MNP crowded out children’s consumption of micronutrient rich foods. Thirdly, we analyse whether our intervention was ineffective because the causes of anaemia amongst our population are fundamentally different from those in populations where similar interventions have been effective. Specifically, we investigate the extent to which childhood anaemia in our population is due to iron deficiency hence determining the extent to which the iron supplementation element of MNP is well-suited to address it.

## The MNP intervention

The MNP we used was ‘Sprinkles’ developed by the Sprinkles Global Health Initiative. Each 1 g sachet, to be sprinkled over a child’s meal daily, contains 12.5 mg iron (ferrous fumerate), 5 mg zinc, 300 µg vitamin A, 30 mg vitamin C and 160 µg folic acid. This safely meets the average child’s daily requirement of iron, Vitamin A, Vitamin C and Folic Acid and the majority of their zinc requirement (World Health Organization, 2005). Our intervention supplemented the children daily for 18 months.

Our intervention and evaluation used the structures of *Familias en Acción* (FeA), Colombia’s conditional cash transfer programme which targets the lowest socio-economic strata (SISBEN level one) which corresponds to roughly the poorest 16% of the population. The MNP intervention was delivered by home visitors who were local women and in 63% of cases were *Madre Líderes*, locally elected female representatives of FeA beneficiaries. Home visitors were paid $15,000 Colombian Pesos (US$40) a month. Before the intervention began, home visitors in MNP intervention areas received a five hour training session, led by a mentor.

The home visitors delivered the appropriate number of sachets to households every fortnight. To minimise the sharing of sachets across children in the household they provided enough sachets for daily consumption of all children younger than six years. During their first visits, home visitors explained to the mothers (or primary carers) how to store and administer the MNP and what to do in the event of side effects. This information was also contained in a booklet that was given to the mothers. Mothers were asked to record daily whether the target child had consumed MNP and, when necessary, why not. In addition to general oversight the mentors managed stock and procurement logistics and compiled the charts of daily MNP intake.

## Methods

### Study design and participants

Our study evaluated the impacts of Psychosocial Stimulation (PS, see Attanasio et al. (2014) for details) and MNP supplementation on child development, alone and in combination. The study design followed a 2×2 clustered, factorial design with four arms: (1) control, (2) PS, (3) MNP, and (4) PS+MNP. While we do not discuss PS or its impacts, described in Attanasio et al. (2014), we account for it fully in the analysis. The interventions were rolled out between February and May 2010 and lasted for 18 months.

The study enroled FeA beneficiaries, who are amongst the poorest 16% of the population, sampled from three geographical regions: (i) Cundinamarca, Boyacá and Santander; (ii) Antioquia, Risaralda and Caldas; and (iii) Huila and Tolima. In each region we selected 32 small towns satisfying the following criteria: (i) FeA had been in operation since it began in 2002; and (ii) the population was between 2000 and 42,000. In each town we randomly selected three *Madre Líders*, after which we identified (through a door-to-door census) all children in the target age range (12–24 months at enrolment) living in families represented by these *Madre Líders*. Amongst these children we randomly selected five for enrolment. The target sample size was, therefore, 1440 (3x32x3×5) children.

### Randomisation and masking

Our study randomly allocated eight towns from each of the three regions to each of the four arms.

For practical reasons we did not use a placebo in areas that did not receive MNP. It was not possible to blind participants to the PS intervention. We made every effort to ensure testers were blind to treatment allocation.

### Outcomes

In analysing the impact of MNP on anaemia our preferred outcome measure is continuous raw haemoglobin (over a binary indicator of anaemia) because of uncertainty over optimal cut-offs and how these should vary with age for young children (Beutler and Waalen, 2006, Brault-Dubuc et al., 1983). However, we also report anaemia for comparability with existing literature. We measured haemoglobin using the HemoCue method, which has been found a suitably accurate method in resource poor settings (Nkrumah et al., 2011). We define anaemia using the WHO cut-off of Hb<110 g/L with WHO altitude adjustments (World Health Organization, 2011b).

We collected socio-economic characteristics and children’s intake of 15 different foodstuffs (selected, through piloting, to include all items common in a Colombian diet) during the past seven days through maternal/carer report in a household questionnaire.

### Statistical analysis

We perform all analysis using STATA (version 12) and adjust all inference (standard errors, confidence intervals and p-values) for clustering at the town level, our unit of randomisation.

#### Validating measurements

We validate our measures of haemoglobin by assessing whether they correlate, assessed by Pearson’s correlation coefficient, with certain characteristics in the anticipated direction (in parentheses): baseline haemoglobin (positive), altitude (positive), age (positive), length-for-age *z*-scores (positive).

#### Power analysis

We analytically calculate the power of our design and regression estimator to detect a positive effect of MNP on haemoglobin concentrations of children in the MNP only group relative to the control, at a 5% significance level, if the true ATE in our population were equal to the weighted mean difference from the RE meta-analysis (5.36 g/L). See Table A2 for analytic formula. In two sensitivity analyses we use (1) the lower bound of confidence interval of estimated ATE from meta-analysis and (2) the standard error from our unadjusted estimate.

#### Effects of MNP on anaemia and haemoglobin

Where haemoglobin is the dependent variable we use linear regression and report adjusted and unadjusted mean differences. Where anaemia is the binary dependent variable we use logistic regression and report adjusted and unadjusted odds ratios. In adjusted measures we control for sex, tester, region, second order polynomials in age and altitude and the baseline value of the dependent variable. We estimate the effect of MNP on haemoglobin for two subsamples, younger children and those with lower haemoglobin measures at baseline using identical methodology.

#### Partial non-compliance analysis (instrumental variables)

We use an instrumental variables approach (Greenland, 2000) to remove confounding factors when we account for partial non-compliance and allow the impact to vary by treatment intensity. In this method we require an instrumental variable that is correlated with MNP consumption but independent of all other factors that affect haemoglobin concentration. In our context, treatment status is the obvious instrumental variable, as it is randomly assigned and therefore uncorrelated with any unobservable determinant of anaemia. It is also highly likely to be correlated with MNP consumption and we test this using an F-test.

#### Analysing the coexistence of anaemia and iron deficiency

To analyse the dynamics of childhood anaemia in Colombia and its relationship with iron deficiency, we use data on haemoglobin and iron deficiency from the second Colombian national survey of nutritional status, ENSIN 2010 (Instituto Colombiano de Bienestar Familiar, 2015). From the ENSIN sample we take children aged 12–42 months (the survey only collected ferritin measurements for children over one year) from the poorest category of Colombia’s six-level categorisation system for determining eligibility for social welfare programmes (SISBEN level one).

We estimate the share of anaemia that could likely be addressed through supplementing children’s diets with iron by looking at the coexistence of anaemia with iron deficiency. We first use multiple imputation (Sterne et al., 2009) to impute missing ferritin measurements. Here we assume that ferritin measurements are missing at random once we control for haemoglobin, height, weight, age, sex, altitude and geographical area. Controlling for these (observable) variables in the imputation routine is important, since missing data (due to refusal for a venous blood sample to be taken) are unlikely to occur at random. Next, we calculate the proportion of anaemic children (Hb<110 g/L, adjusted for altitude) with iron deficiency (ferritin<12 µg/L) on the subsample of children who show no evidence of infection (CRP<1.2 mg/L). We estimate this separately for two sub-samples of the ENSIN survey – those aged 12–24 months (age at baseline in our study) and those aged 30–42 months (age at follow-up in our study).

We also use data on prevalence of anaemia by country from the Demographic and Health Surveys (DHS) Program and PEDNSS (Centers for Disease Control and Prevention, 2015). This enables us to compare the pattern of anaemia we see in Colombia with other countries. When more than one DHS survey is available for a country we use the most recent.

## Results

Of the 1440 children we selected for our study, we excluded from the analysis 11 who were out of the age range. Haemoglobin measurements were taken for all but 33 children at baseline (1396 children). At follow-up, we retained 93.2% of the baseline sample (1332 children) and obtained haemoglobin measurements for 1297. Our sample of analysis consists of the 1296 children for whom we have haemoglobin measurements both at baseline and follow-up.

There is evidence of differential attrition and missing haemoglobin measurements between treatment groups. A significantly higher proportion of the PS group (12.3%) and MNP group (10.2%) than of the control group (5.6%) were either completely lost from the study at follow-up or were missing haemoglobin measurements (*p*<0.05). Nonetheless, attrition and missing haemoglobin are uncorrelated with baseline haemoglobin and other key characteristics. Table 1 shows that our analysis sample were well balanced in terms of key baseline characteristics across treatment and control. Therefore, we do not expect differential attrition to bias our estimates.

**Table 1:** Baseline characteristics.

	**Control**		**MNP+PS**		**PS**		**MNP**		***p*-Value**^a^
Age (months)	18.3	(4.0)	17.9	(3.7)	18.0	(3.8)	17.9	(3.6)	0.785
Male (%)	49.2		50.9		46.1		52.8		0.375
Stunted^b^ (%)	15.8		13.3		13.3		10.8		0.401
Haemoglobin (g/L)	114.5	(12.9)	113.6	(13.1)	112.8	(13.0)	113.7	(12.7)	0.892
Anaemia^c^ (%)	47.4		45.4		48.4		45.6		0.950
Mother’s age (years)	26.1	(7.0)	26.7	(7.7)	26.7	(6.9)	26.0	(6.2)	0.462
Mother’s education (years)	7.4	(3.7)	7.4	(3.5)	6.9	(3.6)	7.3	(3.6)	0.498
Haemoglobin of mother (g/L)	134.0	(14.5)	133.1	(14.0)	131.7	(15.4)	131.6	(14.7)	0.743
Anaemia mother (%)	20.1		22.0		22.5		22.7		0.953
Number of people in household	5.2	(2.3)	5.2	(2.1)	5.4	(2.3)	5.2	(2.1)	0.729

***N***	321		324		308		316		

Standard deviations in parentheses. Significance stars refer to difference in means tests between each intervention group and the control group, accounting for clustering (at the town level): ***p<0.01, **p<0.05, *p<0.1.

a *p*-Values for testing null hypothesis that mean characteristics are equal across all four groups, accounting for clustering at town level.

b Stunting is defined as a *Z*-score height-for-age <−2SD.

c Anaemia is defined in Section “Outcomes”.

### Validity of study design

We calculate the power of our test to detect an average treatment effect (ATE) on haemoglobin equal to an increase in haemoglobin of 5.36 g/L, corresponding to the weighted mean difference (WMD) from the meta-analysis of previous trials (Fig. 1). Using an external benchmark, rather than our own estimated treatment effect, for the ATE under the alternative hypothesis, means our analysis does not fall foul of criticisms relevant to many post-hoc power calculations (Hoenig and Eisey, 2001).

Using our estimated standard errors, we find that the probability that we would have rejected the null of a zero ATE (5% significance level, two-tailed hypothesis test), if the true ATE was equal to 5.36 g/L, is greater than 0.99. In sensitivity analysis we obtain a power of 0.98 and 0.86, respectively, if we use the lower bound of the confidence interval of the estimated WMD (Fig. 1) or if we use the estimated standard error from the unadjusted haemoglobin measurements (all calculations presented in Table A2). Therefore, we can be almost certain that had the true ATE in our population been similar to the mean effect estimated in other studies, we would have detected a positive impact on haemoglobin.

### Validity of haemoglobin measurements

We measured haemoglobin using the HemoCue method which, although not as precise as more expensive alternatives, has been found to be suitably accurate in resource poor settings (Nkrumah et al., 2011). The fieldwork agency checked the machines before and after fieldwork and found that they were functioning satisfactorily. We tested whether individual testers misused the machines by comparing residual haemoglobin measurements between testers, after controlling for important determinants of haemoglobin. We found no evidence of this. In Table A3 we show our haemoglobin measurements are correlated with age, altitude and haemoglobin at baseline in the expected directions. We also found that our measurements are consistent with the ENSIN 2010 data in terms of age and altitude gradients. Overall, there is no evidence that inaccurate haemoglobin readings left us unable to detect a true effect.

### Effect of MNP on anaemia and haemoglobin

The baseline prevalence of anaemia (Hb<110 g/L, adjusted for altitude) was 46.7% in the entire sample and balanced between groups (Table 1). The first panel in Table 2 reproduces the finding of Attanasio et al. (2014) that, at follow-up, the prevalence of anaemia more than halved to 21.0%. However, we see no significant difference between intervention groups. Likewise, mean haemoglobin increased by 7.61 g/L from baseline but there is no difference between groups. The null result remains in our adjusted estimates.

**Table 2:** Intervention Impacts on anaemia and haemoglobin at follow-up, overall and by sub-groups.

	**Control**		**MNP+PS**		**PS**		**MNP**		***p*-Value**^a^
**Full sample**									
**Anaemia**^b^									
%(Fraction)	26.480	(85/321)	18.827	(61/324)	19.481	(60/308)	19.937	(63/316)	
Unadjusted OR (95%CI)^c^			0.644	(0.356, 1.166)	0.672	(0.382, 1.182)	0.691	(0.396, 1.206)	0.419
Adjusted OR (95%CI)			0.678	(0.423, 1.088)	0.766	(0.483, 1.215)	0.677	(0.426, 1.074)	0.304
**Haemoglobin (g/L)**									
Mean(SD)	120.944	(12.065)	122.284	(11.275)	121.305	(11.864)	120.446	(11.874)	
Unadjusted diff (95%CI)			1.340	(−2.288, 4.968)	0.361	(−3.484, 4.207)	−0.498	(−3.994, 2.997)	0.693
Adjusted diff (95%CI)			1.500	(−0.309, 3.308)	1.222	(−0.475, 2.918)	0.272	(−1.607, 2.152)	0.251
***N***	321		324		308		316		

**Anaemic at baseline**^c^									
**Haemoglobin (g/L)**									
Mean(SD)	118.171	(11.411)	119.279	(11.241)	117.852	(11.577)	117.950	(11.666)	
Unadjusted diff (95%CI)			1.108	(−3.027, 5.243)	−0.319	(−4.081, 3.444)	−0.221	(−3.752, 3.310)	0.890
Adjusted diff (95%CI)			0.933	(−1.633, 3.498)	0.651	(−1.673, 2.975)	−0.343	(−2.583, 1.897)	0.634
***N***	152		147		149		144		

**Younger than 18 months at baseline**									
**Haemoglobin (g/L)**									
Mean(SD)	120.905	(11.173)	120.855	(11.816)	120.377	(12.139)	120.206	(12.127)	
Unadjusted diff (95%CI)			−0.050	(−4.626, 4.527)	−0.528	(−5.064, 4.007)	−0.700	(−4.779, 3.380)	0.983
Adjusted diff (95%CI)			0.931	(−1.697, 3.559)	0.676	(−1.689, 3.041)	0.063	(−2.501, 2.626)	0.863
***N***	137		154		138		145		

All ‘adjusted’ quantities adjusted for sex, tester, region, second order polynomials in age and altitude and baseline value of variable using logistic regression for anaemia and linear regression for haemoglobin. Confidence intervals and p-values adjusted for clustering at the town level.

a *p*-Value for null that (i) for anaemia: the ORs are one for all intervention groups vs. the alternative that they are not all one or (ii) for haemoglobin: the mean difference is zero for all intervention groups vs. the alternative that they are not all zero.

b Anaemia is defined in Section “Outcomes”.

c OR=Odds Ratio, CI=Confidence Interval.

**Fig. 2:**
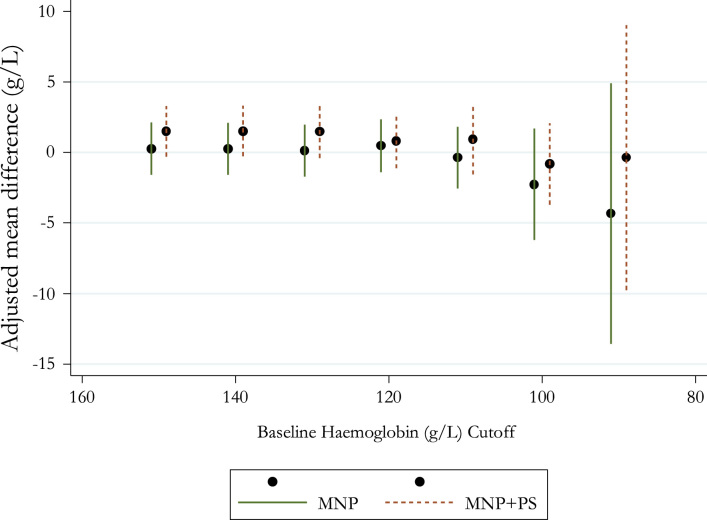
Coefficient plot of estimated effects of MNP on haemoglobin for subgroups of children with baseline haemoglobin below successively lower thresholds. For each baseline haemoglobin cut-off on the *x*-axis the plot shows the estimated effect of MNP on follow-up haemoglobin for the subgroup of children whose baseline haemoglobin was below that cut-off. Points represent point estimates and bars represent the 95% confidence interval around each estimate. All estimated effects adjusted for sex, tester, region, second order polynomials in age and altitude and baseline haemoglobin using linear regression. Confidence intervals adjusted for clustering at the town level.

We might expect to find larger impacts for two subgroups of children: (i) children who were anaemic at baseline and (ii) younger children (since WHO recommends supplementation from 6 months and younger children have higher rates of anaemia). However, the second and third panels in Table 2 show no evidence of positive impacts for either subgroup. Further, in Fig. 2 we look for the existence of threshold effects, where MNP might have been effective for children with baseline haemoglobin below a certain threshold. We plot our estimated effects of MNP and MNP+PS on haemoglobin at follow-up for those children whose baseline haemoglobin fell below successively lower thresholds (adjusted for altitude). We see no evidence that there is any threshold below which MNP has a significant positive effect. If anything, the point estimates become increasingly smaller and then negative for children with the lowest haemoglobin at baseline. This subgroup analysis suggests that, in addition to seeing no average benefit of MNP in our full sample, we also see no evidence of any benefit for the two groups whom we might have expected it to be most effective for.

One possibility that we cannot exclude is that the decline in anaemia we observe between 12–24 months and 30–42 months occurred faster in the MNP treatment groups. We only observe the children 18 months after baseline, when the anaemia rate has already converged to a low level in the overall population. However, if the MNP had had sizeable impacts on the timing of the transition to a low anaemia rate, we might have expected to observe impacts on other outcomes such as height, weight, cognitive, language development 18 months after baseline, which we did not (Attanasio et al., 2014).

### High adoption and compliance

The supplement intake sheets completed by mothers/carers suggest adoption of and compliance with the intervention was high. Table 3 shows that 92% of targeted children adopted the intervention (they consumed some supplements). This 92% consumed a mean (median) of 72.5% (81.3%) of expected consumption. Overall, 68% of targeted children consumed more than 12-months-worth of supplements. Given our intervention provided MNP for longer than similar interventions (18 months compared to the 2–12 months in the studies summarised in Table A1), it is unlikely our null result is due to children consuming insufficient supplements to detect an effect had MNP been efficacious in this population.

**Table 3:** Reported MNP consumption for targeted children.

	**MNP**		**MNP+PS**		**Total**	
	***n***	**%**	***n***	**%**	***n***	**%**
Less than 6 months worth (1–180 sachets)	38	11.66	51	15.27	89	13.48
6–12 Months’ worth (181–360 sachets)	57	17.48	66	19.76	123	18.64
12–18 Months’ worth (361–546 sachets)	206	63.19	162	48.50	368	55.76
More than expected consumption (>546 sachets)	25	7.67	55	16.47	80	12.12
**Total adoption**	326	91.32	334	92.78	660	92.05

We adopt an instrumental variables approach (described in Section “Partial non-compliance analysis (instrumental variables)”) to allow for partial non-compliance and for the effect on haemoglobin concentration to vary with MNP intake. From Table A4 we see that treatment status is a strong instrument for supplement intake (i.e. treatment status is highly correlated with intake) (*F*-statistic=334.37, *p*=0.000). However, the estimated effect of MNP on haemoglobin remains insignificant and close to zero (*p*=0.733). This suggests the null result in the intention-to-treat analysis is not due to incomplete adoption and low compliance.

Since intake records are based on maternal report, a concern might be that mothers over-reported consumption if they feared non-compliance could affect their permanence in the intervention or FeA more generally. However, one indication that this did not occur is that mothers in the MNP-only group reported significantly higher consumption (mean=403 sachets) than mothers in the MNP+PS group (mean=388 sachets) (*p*<0.05). Since anecdotal and qualitative evidence suggest that mothers greatly valued the PS intervention, this suggests that they did not over-report for fear of losing access to other benefits. In addition, self-reported data was corroborated by home visitors collecting used sachets. Mentors compared the two sources while processing the intake sheets and found they coincided the vast majority of the time.

### No dietary substitution

Our study design did not use a placebo. Therefore it is possible that parents altered their children’s diet in response to MNP. For example, parents in the MNP groups may have reduced the quantity of iron rich food since MNP contained iron, a phenomenon reported by Murphy and Allen (2003), thereby mitigating the effect of MNP. However, analysis of the extensive child food consumption data shows no evidence of substitution away from iron rich foods in the MNP groups *vis-à-vis* the control group (Table 4).

**Table 4:** Follow-up consumption of foodstuffs related to iron intake and absorption – number of days out of the last seven the child consumed the listed food.

	**Control**		**MNP+PS**		**PS**		**MNP**	
**Haem (highly absorbable) iron rich foods**								
Beef, liver etc.	3.643	(2.255)	3.796	(2.216)	3.638	(2.300)	3.838	(2.306)
Poultry	1.715	(1.470)	1.859	(1.424)	1.791	(1.565)	2.091*	(1.613)
Swine, rabbit, lamb	0.279	(0.813)	0.411	(1.078)	0.494	(1.254)	0.298	(0.770)
Fish	0.520	(1.052)	0.524	(0.910)	0.553	(1.025)	0.579	(1.046)

**Non-haem iron rich foods**								
Pulses (fresh)	1.492	(1.958)	1.473	(1.759)	1.741	(2.028)	1.042*	(1.491)
Pulses (dried)	2.508	(1.858)	2.611	(1.812)	2.616	(1.900)	2.634	(1.853)
Bienestarina	2.912	(2.688)	2.900	(2.669)	2.837	(2.595)	3.123	(2.754)

**Foods that may impair iron non-haem absorption**								
Rice	4.837	(2.399)	5.310	(2.275)	4.737	(2.327)	4.877	(2.313)
Bakery products and maize	4.730	(2.349)	4.821	(2.354)	4.800	(2.287)	4.696	(2.378)
Eggs	4.618	(2.349)	4.931	(2.271)	4.650	(2.181)	4.482	(2.216)
Milk or dairy products	6.342	(1.629)	6.179	(1.751)	6.125	(1.831)	6.314	(1.598)

**Foods that may aid non-haem iron absorption**								
Potatoes, plantains etc.	5.254	(2.091)	5.448	(2.027)	5.353	(2.155)	5.184	(2.123)
Pumpkin, carrot, peppers, spinach	3.154	(2.321)	3.292	(2.495)	3.525	(2.487)	3.485	(2.387)
Fruits or juices	4.966	(2.403)	5.373	(2.239)	5.206	(2.333)	5.133	(2.223)

***N***	319		319		320		309	

Standard deviations in parentheses. Significance stars refer to difference in means tests between each intervention group and the control group, using cluster robust standard errors: ***p<0.01, **p<0.05.

⁎ *p*<0.1. Refers to difference in means tests between each intervention group and the control group, using cluster robust standard errors.

### Coexistence of anaemia and iron deficiency

In our analysis of the ENSIN sample (described in Section “Analysing the coexistence of anaemia and iron deficiency”), we find that of the 38.9% of 12–24 month olds (the age of our study children at baseline) who are anaemic, 34.0% have iron deficiency, typically considered to be the largest cause of anaemia. This suggests that iron supplementation through MNP has the potential to reverse anaemia through its iron supplementation element in a relatively low proportion of the ENSIN sample of 12–24 month olds – just the 13.2% who have both iron deficiency and anaemia. When we repeat this analysis for the older ENSIN children (30–42 months) we find that, in addition to the overall prevalence of anaemia falling to 19.0%, the proportion of this anaemia that co-exists with iron deficiency falls substantially to 15.1%. This suggests that, by 30–42 months, the proportion of children in the ENSIN sample whose anaemia status could plausibly be improved by the iron supplementation element of MNP is just 2.9%.

## Discussion

The results presented in Sections “Validity of study design” to “No dietary substitution”, Sections “Validity of study design” to “No dietary substitution”, Sections “Validity of study design” to “No dietary substitution”, Sections “Validity of study design” to “No dietary substitution”, Sections “Validity of study design” to “No dietary substitution” suggest that for Colombian children aged 12–24 months from poor backgrounds and living in small towns, MNP supplementation is ineffective when delivered in real world conditions and these analyses suggest that this was due to a lack of efficacy. This finding holds even for children we might have expected to benefit the most – younger children and those who were anaemic at baseline. Key to understanding this, when similar interventions have been successful, is the progression of anaemia, and its causes, with age in our population.

**Fig. 3:**
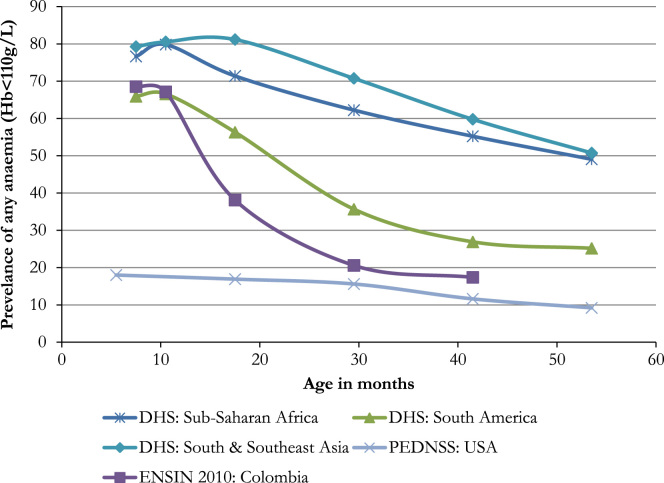
Prevalence of childhood anaemia by age across different regions. Colombian data from ENSIN 2010 for sub-sample SISBEN level one households. USA data from PEDNSS. Continent level data from all DHS surveys which collected childhood anaemia rates. Most recent data used when more than one survey available. Country data weighted by World Bank population estimates of children under four to estimate the combined prevalence, grouped at the continent level. Countries included in continent level averages are listed in the online appendix (Table A5).

Nearly 70% of the poorest Colombian children aged 6–12 months are anaemic (Fig. 3), just 10 percentage points below rates in South and Southeast Asia and Sub-Saharan Africa. However, while anaemia appears very persistent in the Asian and African countries, in Colombia, the rate falls very quickly, almost halving in seven months and falling to 20%, a rate comparable to the USA, by 30 months. This trend appears similar to, but more pronounced than, other countries in South America; and is very similar in the evaluation sample we have analysed.

This pattern suggests that we targeted children who were already too old to receive maximum benefit from MNP. From Fig. 1 we notice that the studies that found MNP to be most effective at increasing haemoglobin were those with very low average initial haemoglobin (severe anaemia) and where the control group still had very low levels at follow-up (persistent anaemia). In contrast, at the time of our baseline survey mean haemoglobin in our study population was the highest amongst all the studies listed in Fig. 1 and mean haemoglobin in the control group increased rapidly over the duration of the intervention, from 114.5 g/L to 120.9 g/L. The increase was even more dramatic for those controls that were anaemic at baseline (104.2–118.2 g/L). This supports the idea that our study began after and continued during a period of rapid improvement in anaemia and so did not act in time to address the nearly 70% of 6–12 month olds in Colombia (Fig. 3) who are anaemic.

In Section “Coexistence of anaemia and iron deficiency” we show that just over a third of anaemia co-exists with iron deficiency for children aged 12–24 months (the baseline ages for our study children) and this falls to just 15.1% by ages 30–42 months (the follow-up ages of our study children), in a comparable sample of Colombian children. This proportion is substantially lower than in most settings where MNP has been successful (see Table 5). We interpret this as implying that although iron deficiency is associated with childhood anaemia in our population at the start of our intervention this association is weaker than in other study settings. Therefore coupled with lower absolute rates of anaemia, the proportion of study children for whom the iron supplementation element of MNP could plausibly reverse their anaemia is far lower. This proportion falls rapidly over the ages we intervene to 2.9% by the ages where we collected follow-up. Table 5 shows this is in contrast to studies where MNP has been effective. For example, in the study of Soofi et al. (2013) in Pakistan, 62.0% of anaemic children in the control group at follow-up also had iron deficiency resulting in 57.0% having both iron deficiency and anaemia. This contrast makes it unsurprising that we find no effects when others do.

**Table 5:** Co-existence iron deficiency and anaemia in Colombia compared with other MNP studies.

**Paper**		**Prevalence of**		**Proportion of anaemia with iron deficiency (%**)	**Anaemia cut-off**^b^
	**Country**	**Anaemia**	**IDA**^a^		
ENSIN 2010^c^ (12–24 months)	Colombia	38.9%	13.2%	34.0	110
ENSIN 2010 (30–42 months)	Colombia	19.0%	2.9%	15.1	110
Soofi 2013 (FU) (Soofi et al., 2013)	Pakistan	92.0%	57.0%	62.0	120
Jack 2012 (BL) (Jack et al., 2012)	Cambodia	74.7%	55.3%	74.1	100
Jack 2012 (FU) (Jack et al., 2012)	Cambodia	47.3%	25.0%^d^	52.9	100
Macharia-Mutie 2012 (FU) (Macharia-Mutie et al., 2012)	Kenya	38.4%	21.5%	56.0	110
Macharia-Mutie 2012 (FU) (Macharia-Mutie et al., 2012)	Kenya	32.3%	11.8%	36.5	110
Veenemans 2011 (BL) (Veenemans et al., 2011)	Tanzania	68.0%	17.0%	25.0	110
Adu-Afawuah 2007 (BL) (Adu-Afarwuah et al., 2007)	Ghana	26.3%	17.6%	67.1	100
Adu-Afawuah 2007 (FU) (Adu-Afarwuah et al., 2007)	Ghana	32.3%	30.5%	94.4	100

BL=baseline (weighed average of all intervention groups), FU=follow-up (control group only).

a IDA=Iron deficient anaemia (defined as having both iron deficiency and anaemia).

b Anaemia cut-off is haemoglobin concentration (in g/L) below which child is a defined as anaemic. In all studies the cut-off for defining iron deficiency was 12 ng/mL.

c Refers to ENSIN sample described in Section “No dietary substitution”, missing ferritin measures imputed using multiple imputation.

d Figures read off graph.

The high early rates of anaemia that quickly begin to fall after one year, and the relatively weak association between anaemia and iron deficiency that becomes increasingly weak as children get older, brings us to children’s diet. Evidence from our household survey suggests that, in our population even deprived children consume relatively large quantities of meat and other sources of highly absorbable haem iron. At follow-up, children ate beef/liver a mean of 3.77 days out of the past seven days and poultry on 1.86 days (Table 4). This is high relative to children’s diet in Sub-Saharan Africa-our own (unpublished) data from rural Malawi showed only 38% of children had eaten any meat in the past three days. It is likely higher still relative to many areas of South and Southeast Asia. We also observe a relatively high consumption of Bienestarina, a government provided blend of flour and milk powder fortified with iron and other vitamins and minerals (although substantially less than our MNP), amongst our study population. At baseline 65% of children had consumed Bienestarina during the last week. Similarly, our study children were not eating unusually large quantities of foods with high phytate or phenolic contents which inhibit iron absorption. Therefore, descriptives of food intake render it unlikely that anaemia in Colombian children older than one year, is predominantly caused by current dietary factors, meaning it is unlikely that MNP supplementation could be useful in this context.

If the anaemia we observe after one year of age is largely unrelated to current micronutrient intake, why are nearly 70% of 6–12 month olds in Colombia, from the bottom socio-economic strata, anaemic? This study was not designed to answer this question and we provide no firm evidence here. However, we raise some potential hypotheses and provide correlation evidence (controlling for age) from under 12 month olds in the ENSIN sample that is consistent with such hypotheses.

A first hypothesis relates to early feeding practices. In our ENSIN sample we find that mean haemoglobin was substantially (5.06 g/L) higher in infants who were introduced to semi-solid foods between 6 and 8 months (consistent with WHO guidelines) than infants for whom semi-solids had been introduced prior to 6 months. This is consistent with an argument that when semi-solids are introduced before 6 months these are often foods not rich in iron and therefore, because they reduce the consumption of breast milk, they reduce overall iron intake (Geissler & Powers, 2010). Similarly, dietary diversity and meal frequency in the early weaning period appear correlated with haemoglobin. Infants that met the WHO criteria for a ‘minimum acceptable diet’ over the past 24 hours had, on average, a haemoglobin of 1.58 g/L higher than those who did not. Work by Olaya, Lawson, and Fewtrell (2013) also suggests that dietary diversity and micronutrient intake during the early weaning period is key to childhood anaemia in Colombia. Their RCT found that a 6 month intervention encouraging parents to continue breastfeeding, offer red meat more than 3 days a week and offer fruit and vegetables daily increased haemoglobin of 6 month olds. Thus, while we find that diet after 12 months does not contribute greatly to high rates of early childhood anaemia in Colombia it may be that the micronutrient content of children’s diet during the early weaning period is crucial.

The second hypothesis relates to infants’ iron stores at birth, which form infants’ primary source of iron for the first months of life. Iron status of mothers during pregnancy is a key determinant of infant iron stores at birth (Allen, 2000). In our ENSIN sample we find that infants (6–12 month olds) whose mothers had taken iron supplements for 4 or more months of pregnancy had a mean haemoglobin concentration 3.10 g/L higher than those whose mothers had not. This is consistent with inadequate iron statuses of pregnant mothers being a key cause of early childhood anaemia in Colombia. A related potential factor is early clamping of the umbilical cord which is an important determinant of low iron stores in infants (McDonald, Middleton, Dowswell, & Morris, 2013). While we do not have access to reliable information on timing of cord clamping in Colombia this may be important in analysing early childhood anaemia in Colombia.

## Conclusions

Although the population of Colombian infants we studied fitted the criteria of the WHO guideline for universal MNP supplementation, they gained no tangible benefit from the intervention in terms of increased haemoglobin. This suggests that favourable evidence from other settings on MNP as a population wide intervention for children 12–24 months is not transferable to a Colombian context. We suggest that this is likely due to the causes of childhood anaemia, and its age profile, being different to those settings. Given the ineffectiveness and likely inefficacy of MNP administered from 12 months of age, and the pattern of anaemia we see in Colombia and other countries with a similar profile, future research might usefully study the effects of earlier interventions (gestation to 12 months) targeting early feeding practices and infant iron stores.
