# Supplementary material for: Why is multiple micronutrient powder ineffective at reducing anaemia among 12–24 month olds in Colombia? Evidence from a randomised controlled trial
Source: SSM Popul Health. 2016 Mar 7;2:95–104. doi: 10.1016/j.ssmph.2016.02.004 (PMC5757801; doi:10.1016/j.ssmph.2016.02.004)
Supplement: Supplementary file 1 — Supplementary material [file mmc1.docx]

# Appendices

### Table A1: Summary of MNP studies included in meta-analysis

| Study | Region, Country | Study design | Target Population | Intervention (sample size) | Control (sample size) |
| --- | --- | --- | --- | --- | --- |
| Attanasio et al. 2014 [2] | Colombia | Cluster RCT | 12-24 months at baseline from Familias en Accion (CCT) recipients (the bottom socio-economic strata). | MNP containing 12.5mg ferrous fumerate. (n=652, 48 clusters) | No intervention (n=644, 48 clusters) |
| Zlotkin et al. 2013 [13] | Ghana | Cluster RCT | 6 to 35 month olds living in rural central Ghana with Hb>70g/L and not severely wasted. Mean baseline haemoglobin was 103g/L. | MNP containing 12.5mg ferrous fumerate. (n=967, 780 clusters) | MNP without iron (n=967, 772 clusters) |
| Soofi et al. 2013 [14] | Sindh, Pakistan | Cluster RCT | 6 to 18 month olds from both rural and urban site in Sindh. Mean haemoglobin at baseline was 105g/L and around 20% of children had iron deficiency anaemia. | MNP (containing 12.5mg ferrous fumerate) (n=889, 85 clusters, 210 Hb measurements) | No intervention (n=947, 85 clusters, 211 Hb measurements) |
| Jack et al. 2012 [15] | Svay Rieng Operational Health District, Cambodia | Cluster RCT | 6 month olds from a rural district of Cambodia. Around 47% of children were classified as anaemic (Hb<100g/L) at baseline. | MNP (containing 12.5mg ferrous fumerate) with a feeding education programme (n=675, 10 clusters) | Feeding education programme only (n=675, 10 clusters) |
| Suchdev et al. 2012 [16] | Nyando Division, Kenya | Cluster RCT | 6 to 23 month old children from the rural Nyando Division. Around 65% of children were classified as anaemic (Hb<110g/L) at baseline. | Commercial vendors marketed and sold MNP over a 12 month period (n=721, 30 clusters, 427 Hb measurements) | No intervention (n=699, 30 clusters, 407 Hb measurements) |
| Macharia-Mutie et al. 2012 [17] | Mwingi District, Kenya | RCT | 12 to 59 month old preschool children from Mwingi District (semi-arid rural areas). Around 38% were anaemic (Hb<110g/L) at baseline. | Unrefined maize porridge with MNP on schooldays for 16 weeks (n=93) | Unrefined maize porridge without MNP (n=93) |
| Kounnavong et al. 2011 [18] | Lao People's Democratic Republic | RCT | 6 to 52 month olds in six rural communities of Lao PDR. Around 50% were classified as anaemic (Hb<100g/L) at baseline. | MNP (containing 10mg iron) once daily for 24 weeks(n=111) | Given high dose vitamin A supplementation (n=110) |
| Veenemans et al. 2011 [19] | Handeni District, Tanzania | RCT | 6 to 60 month olds in a rural area of Handeni District. Around 70% of children were classified as anaemic (Hb<110g/L) at baseline | MNP to be mixed with clean water or breast milk (containing 18mg ferrous fumerate) once daily (n=151) | Placebo powder (n=153) |
| Lemaire et al. 2011 [20] | Bangladesh | RCT | 12 to 24 month olds with moderate to severe malnutrition (weight-for-age z score ≤ -2) and haemoglobin concentrations of between 70g/L and 110g/L | Iron MNP (containing 12.5mg ferrous fumerate) once daily for two months (n=136) | Placebo powder (n=132) |
| Lundeen et al. 2010 [21] | Kyrgyz Republic | Cluster RCT | 6 to 36 month year olds from rural districts in the Kyrgyz Republic.  72% were classified as anaemic (Hb<110g/L) at baseline. | MNP (including 12.5mg ferrous fumerate) daily for two months (12 clusters, n=1103, 947 Hb measurements) | No intervention (12 clusters, n=1090, 922 Hb measurements) |
| Adu-Afarwuah 2007 [22] | Koforidua, Ghana | RCT | 6 month old infants receiving any breast milk. Around 25% were classified as anaemic (Hb<100g/L) at baseline. | MNP (including 12.5mg ferrous fumerate) daily for six months (n=98, 98 Hb measurements) | No intervention (n=96, 96 Hb measurements) |
| Menon et al. 2007 [23] | Haiti | Cluster RCT | 12 to 27 month olds living in rural Haiti who were receiving wheat-soy-blend ration with Hb>70g/L. 46% of the children were classified as anaemic (Hb<100g/L) at baseline. | MNP (including 12.5mg ferrous fumerate) daily for two months (n=254, 6 clusters, 244 Hb measurements) | No intervention (still received the wheat-soy-blend (n=161, 4 clusters, 154 Hb measurements) |
| Sharieff et al. 2006 [24] | Karachi, Pakistan | RCT | 6 to 12 month olds from an urban slum of Karachi who had had at least one episode of diarrhoea in the past two weeks | MNP (including 30mg ferrous fumerate) daily for two months (n=22, 13 Hb measurements) | Placebo powder (n=25, 13 Hb measurements) |
| Giovannini et al. 2006 [25] | Kompong Chhnang Province, Cambodia | RCT | 6 month old infants living in the Tuk Phos (agricultural) district with Hb>70g/L. Around 80% of infants were classified as anaemic (Hb<110g/L) at baseline. | MNP (including 12.5mg ferrous fumerate), daily for 12 months (n=68, 65 Hb measurements) | Placebo powder (n=68, 60 Hb measurements) |

We include all published randomised trials found through an extensive but non-systematic search of literature published up to and including November 2015, which compare MNP with iron to no intervention, or a placebo, or MNP without iron, in populations of children under two years at the start of the intervention.

## Table A2: Power analysis

|  | **X: External benchmark of ATE** | **se: Standard error of our ATE estimate** | **Power** |
| --- | --- | --- | --- |
| **Main power calculation** | 5.36g/L | 0.95g/L | >0.99 |
| **Sensitivity analysis 1** | 3.82g/L | 0.95g/L | 0.98 |
| **Sensitivity analysis 2** | 5.36g/L | 1.76g/L | 0.86 |

‘Power’ refers to the power of our design and regression estimator to detect a positive effect of MNP on haemoglobin concentrations of children in the MNP only group relative to the control, at a 5% significance level, if the true ATE in our population were equal to the external benchmark, X. This is calculated analytically and is equal to $\text{Φ(}\frac{\text{X-1.96se}}{\text{se}}\text{)}$. In the ‘Main power calculation’ the external benchmark is the overall WMD of a RE meta-analysis of MNP studies (excluding our own (see Figure 1) and the standard error is the standard error on our adjusted estimate of the difference in mean haemoglobin between the control group and the MNP only group. In ‘Sensitivity analysis 1’ we use the lower bound of confidence interval of estimated ATE from meta-analysis and in ‘Sensitivity analysis 2’ we use standard error from estimate not adjusting for controls.

**Table A3: Correlations with haemoglobin (g/L) measurements**

|  | | **Correlation co-efficient** |
| --- | --- | --- |
| Haemoglobin at Baseline (g/L) | 0.339*** |  |
| Altitude (km) | 0.284*** |  |
| Age (months) | 0.056** |  |
| Length-for-Age z-score | 0.065** |  |
|  |  |  |
| Observations | 1,127 |  |

Significance stars refer hypothesis test that the correlation coefficient is equal to zero, adjusting for clustering at the town level: *** p<0.01, ** p<0.05, * p<0.1*

Table A4: Instrumental variable estimate of the effect of MNP intake intensity on haemoglobin (g/L) measurements

| **I-V/2SLS coefficient (s.e.) on Total supplement intake (x1000)** | 0.582 |
| --- | --- |
|  | (1.708) |
|  |  |
| **N** | 1,269 |
| **F-stat for instruments in first stage** | 334.37 |

Total supplement intake is instrumented by treatment allocation (i.e. dummy variables for the three intervention groups). Cluster robust (at town level) standard errors in parentheses. Significance stars refer hypothesis test that coefficient is equal to zero, adjusting for clustering at the town level: *** p<0.01, ** p<0.05, * p<0.1*. Both regressions control for full set of controls (sex, tester, region, second order polynomials in age and altitude and baseline haemoglobin).

Table A5: Countries included in continent level averages for Figure 3

| Sub-Saharan Africa | Angola, Benin, Burkina Faso, Burundi, Cameroon, Democratic Republic of Congo, Cote d'Ivoire, Ethiopia, Gabon, Ghana, Guinea, Lesotho, Madagascar, Malawi, Mali, Mozambique, Niger, Rwanda, Sao Tome and Principe, Senegal, Sierra Leone, Swaziland, Tanzania, Uganda, Zimbabwe, |
| --- | --- |
| South and South-east Asia | Bangladesh, Cambodia, India, Nepal, Timor-Leste, |
| Latin America | Bolivia, Guyana, Haiti, Honduras, Peru. |
